# Increased risk of brain metastases among patients with melanoma and PROM2 expression in metastatic lymph nodes

**DOI:** 10.1002/ctm2.198

**Published:** 2020-12-02

**Authors:** Thuy Thi Nguyen, Guillaume Gapihan, Pauline Tetu, Frédéric Pamoukdjian, Morad El Bouchtaoui, Christophe Lebœuf, Jean‐Paul Feugeas, Justine Paris, Barouyr Baroudjian, Julie Delyon, Samia Mourah, Céleste Lebbé, Anne Janin, Guilhem Bousquet, Maxime Battistella

**Affiliations:** ^1^ INSERM U942 Universités de Paris et Sorbonne Paris Nord Bobigny France; ^2^ AP‐HP Hôpital Avicenne Oncologie Médicale Bobigny France; ^3^ Medical Oncology Department A National Cancer Hospital Hanoi Vietnam; ^4^ Hôpital Saint‐Louis Université de Paris Paris France; ^5^ Dermatologie AP‐HP Hôpital Saint Louis Paris France; ^6^ Médecine Gériatrique AP‐HP Hôpital Avicenne Bobigny France; ^7^ Université Sorbonne Paris Nord, Cardiovascular Markers in Stressed Conditions, MASCOT Bobigny France; ^8^ INSERM U722 Paris France; ^9^ Université de Franche‐Comté Besançon France; ^10^ INSERM U976 Paris France; ^11^ Solid Tumor Genomic Department AP‐HP, Hôpital Saint Louis Paris France; ^12^ Laboratoire de Pathologie AP‐HP Hôpital Saint‐Louis Paris France

Dear Editor,

Brain metastases occur in the progression of metastatic melanoma in up to 44% of cases.^1^ Despite evidence of clinical benefit of combined immunotherapies on melanoma brain metastases,^2^ more than 50% of patients will have brain progression, challenging daily practice in oncology. Molecular markers predictive of the risk of melanoma brain metastases remain largely to be identified.^3^

In our study, we performed transcriptomic analyses on laser‐microdissected tumor cells from metastatic lymph nodes of patients with melanoma, to identify biomarkers associated with the occurrence of brain metastases over a median follow‐up of 48 months. We followed REMARK recommendations for tumor marker prognostic studies.^4^ All methods are fully detailed in Material and Method in the Supporting Information.

Among the 51 patients selected for the development cohort (Figure S1), after a median follow‐up of 48 months from the time of regional lymph node disease, 19 (37%) developed brain metastases (Group 3), whereas 32 (63%) did not (Group 1 including patients with only regional lymph node metastases and who did not relapse, and Group 2 including patients without brain metastases but with other metastatic localizations) (Table S1 for patients’ characteristics and Figure 1A). The median overall survival calculated from first diagnosis of regional lymph node metastasis was significantly shorter among patients with brain metastases than among patients without (39 vs 76 months, *P* < .01) (Figure S2). The median survival from the time of brain metastases was 13.3 months (range: 2‐72 months).

**FIGURE 1:**
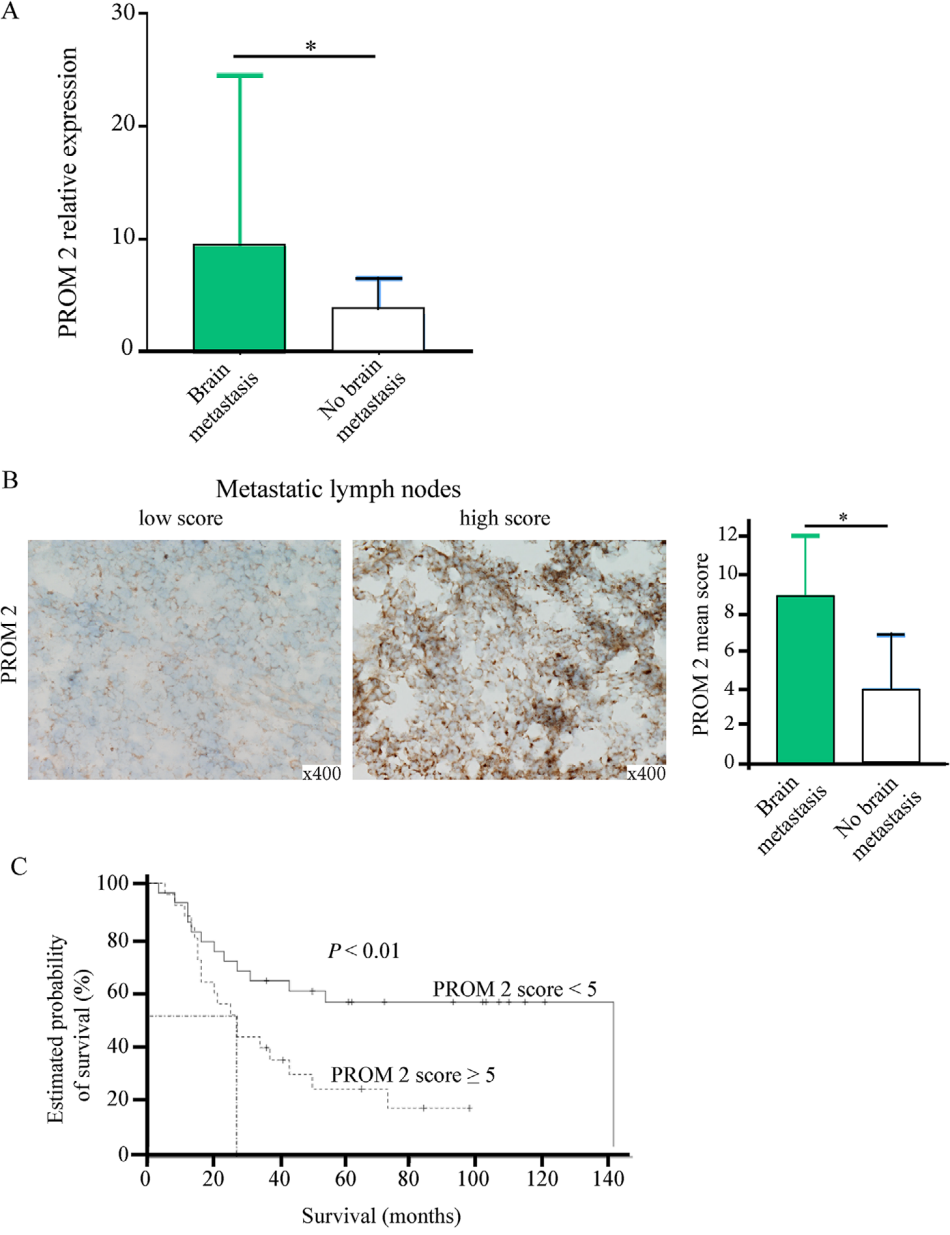
*PROM2* mRNA expression, “PROM2 IHC score” in metastatic lymph nodes, and survival data in the development cohort. (A), *PROM2* mRNA expression is significantly higher in metastatic lymph nodes from patients with brain metastases than in those from patients without brain metastases (*P* < .05). (B), Using immunostaining on metastatic lymph nodes, the mean “PROM2 IHC score” is significantly higher among patients with brain metastases than among those without (*P* < .05). (C), Survival according to the “PROM2 IHC score” level in the development cohort of 51 patients. A “PROM2 IHC score” ≥5 is significantly associated with a shorter survival

Each metastatic lymph node was laser microdissected to select a minimum number of 1500 tumor cells. After RNA extraction, all samples had a RNA integrity number over 7, enabling transcriptomic analyses. On transcriptomic data, multivariate analysis was carried out to compare patients with and without brain metastases. We focused on the *PROM2* gene, also called prominin‐2, with some of the highest *d*‐scores at 4.6 and fold change of 3.3 (Table S2), and because PROM2, a membrane glycoprotein and a second member of the prominin family, induces membrane protrusions^5^ and could thus be implicated in invasive processes. Using reverse transcription‐polymerase chain reaction (RT‐qPCR), the median expression of *PROM2* mRNA was significantly higher in metastatic lymph nodes from patients who subsequently developed brain metastases (Δ cycle threshold [Ct] = 4.9, interquartile range [IQR] = 6.3 vs ΔCt = 2.1, IQR = 3.9; *P* = .005; Figure 1A).

We then analyzed transcriptomic data downloaded from three public databanks (the Cancer Genome Atlas SKCM and GSE22155 and GSE65904 cohorts^6^, ^7^, ^8^), and found that a high *PROM2* expression in melanoma metastatic lymph nodes was associated with poor survival (Figure S3).

Using immunohistochemistry (IHC) on the 51 metastatic lymph nodes of the development cohort, PROM2 was only expressed by cancer cells and the mean “PROM2 IHC score” was significantly higher among patients with brain metastases compared to patients without (8.8 vs 4; *P* < .01) (Figure 1B). Overall survival was significantly longer among patients who had a “PROM2 IHC score” <5 than among patients who reached the cutoff of 5 (*P* < .01; Figure 1C).

Across the three groups of patients, we observed a gradual, significant increase in PROM2 mRNA and protein expression from Group 1 to Group 3 (Figure 2B). After a median follow‐up of 80 months, overall survival was also much longer for Group 1 with only metastatic regional lymph nodes (Figure 2C).

**FIGURE 2:**
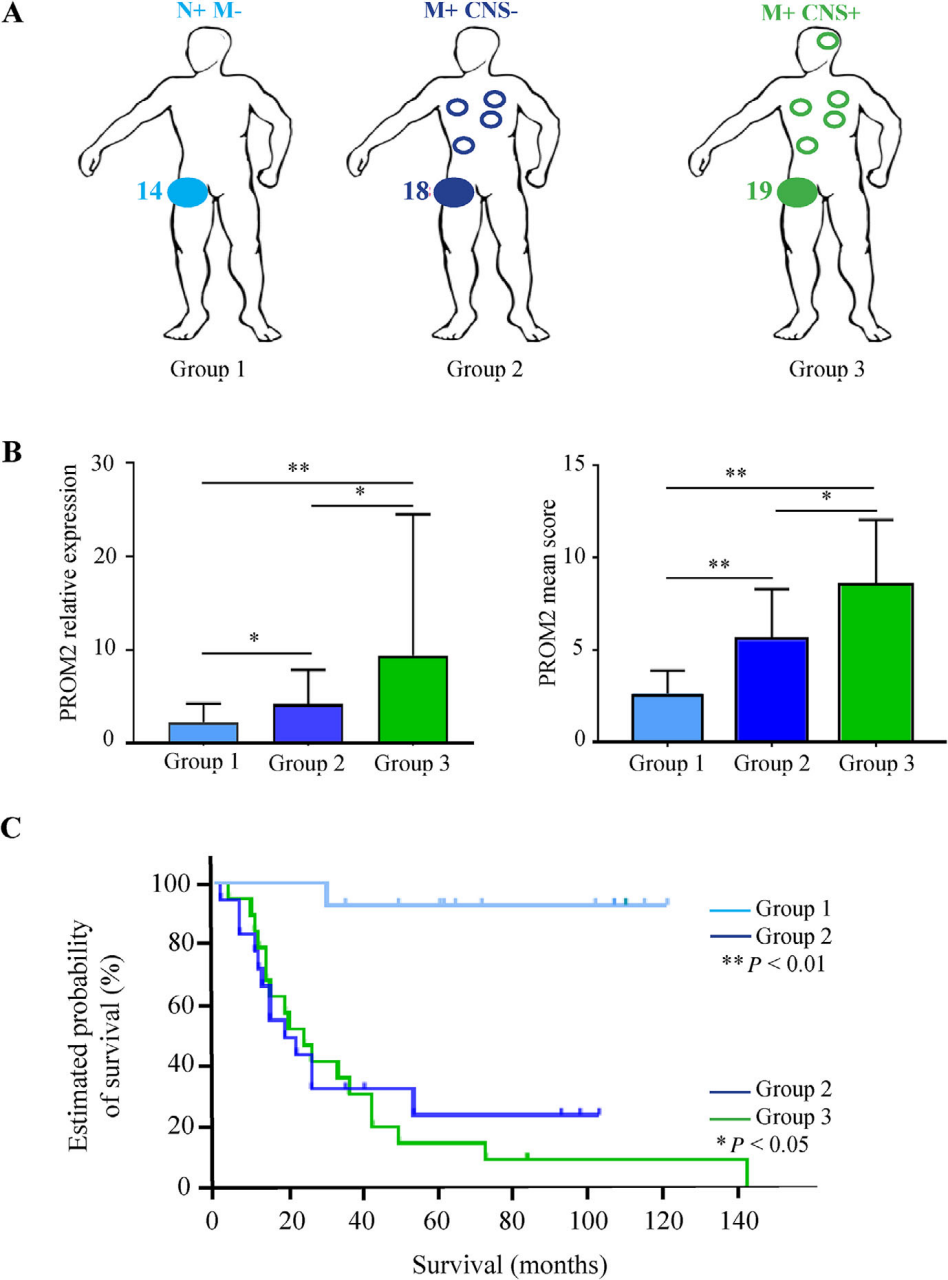
PROM2 expression and survival data according to subgroups in the development cohort. (A), Group 1 includes patients with only regional lymph node metastases; Group 2 includes patients without brain metastases but with other distant metastatic localizations; Group 3 includes patients with brain metastases. (B), *PROM2* mRNA expression and PROM2 mean score in the three different groups. *PROM2* mRNA expression and PROM2 mean score are gradually and significantly different in the three groups. (C), Survival curves according to the three different groups. *P < .05; **P < 0.01

Using multivariate regression, a “PROM2 IHC score” ≥5 (odds ratio at 28.2) and the presence of bone metastases were the two variables significantly associated with the risk of brain metastases (Table S3).

Between 2013 and 2014, 50 additional patients with stage III melanoma at diagnosis and a frozen biopsy sample from lymph node metastases were included in the validation cohort. After a median follow‐up of 48 months from the time of the regional lymph node disease, 19 patients (38%) developed brain metastases, whereas 31 (62%) did not. There was no significant difference between the development and the validation cohorts (Table S4).

In this validation cohort, when *PROM2* mRNA expression was assessed on melanoma cancer cells laser microdissected from metastatic lymph nodes, it was significantly higher among patients who developed brain metastases (median ΔCt = 5.1, IQR = 2.3 vs ΔCt = 2, IQR = 4.6) (*P* < .01). The “PROM2 IHC score” was also significantly higher in case of brain metastases (7.4 vs 2.1; *P* < .01). Using a cutoff of ≥5 for the “PROM2 IHC score” in multivariate regression, the “PROM2 IHC score” was the only factor associated with the risk of brain metastases.

In the two cohorts, in univariate analysis, a high “PROM2 IHC score” ≥5 in metastatic lymph nodes was not associated with the risk of other metastatic sites (lung, liver, and bone), except for lung metastases in the validation cohort (Table S2). In both the development and validation cohorts, the presence of brain metastases and a “PROM2 IHC score” of ≥ 5 were the only two factors significantly associated with mortality (Table 1; Figure S4).

**TABLE 1:** Univariate and multivariate analyses of factors associated with mortality in the development and validation cohorts

	Development cohort (n = 51)				Validation cohort (n = 50)			
Variables	Univariate analysis HR [95% CI]	*P*‐value	Multivariate analysis aHR [95% CI]	*P*‐value	Univariate analysis HR [95% CI]	*P*‐value	Multivariate analysis aHR [95% CI]	*P*‐value
Age (years), mean ± SD	1.01 [0.98‐1.03]	.37			0.98 [0.96‐1.01]	.26		
Gender (women)	0.94 [0.46‐1.93]	.88			0.82 [0.39‐1.75]	.62		
Initial TNM classification		.01		–		.94		
IIIB	1 (reference)		–		1 (reference)			
IIIC	1.22 [0.57‐2.60]		–		1.12 [0.53‐2.34]			
IIID	7.26 [1.85‐28.4]		–		0.86 [0.11‐6.58]			
Primary site of melanoma		.49				.20		
Head and neck	1 (reference)				1 (reference)			
Trunk	0.91 [0.26‐3.15]				0.42 [0.10‐1.64]			
Upper limb	0.48 [0.13‐1.75]				0.80 [0.19‐3.26]			
Lower limb	0.46 [0.15‐1.44]				1.08 [0.30‐3.86]			
Unknown	0.49 [0.05‐4.45]				–			
Metastatic site								
Brain	2.61 [1.28‐5.34]	.008	–	–	5.20 [2.39‐11.3]	<.0001	8.08 [2.49‐26.2]	.0005
Lung	3.82 [1.78‐8.18]	.0005	10.7 [2.86‐39.9]	.0004	2.36 [1.13‐4.96]	.02	–	–
Bone	2.00 [0.91‐4.36]	.08	–		2.50 [1.17‐5.37]	.01	–	–
Liver	1.85 [0.90‐3.79]	.09	–	–	2.07 [0.99‐4.31]	.05	–	–
Breslow index (mm), per 1 IQR of more	1.19 [1.04‐1.36]	.01	–	–	0.93 [0.82‐1.06]	.28		
Ulceration (yes)	2.75 [1.26‐6.00]	.01	–	–	1.19 [0.53‐2.65]	.67		
BRAF status:								
BRAF V600E (yes)	1.49 [0.73‐3.04]	.26			1.49 [0.65‐3.42]	.34		
*PROM2* mRNA expression, per 1 IQR of more	1.02 [0.99‐1.05]	.11	–	–	1.16 [1.03‐1.31]	.01	–	–
PROM2 IHC score								
High (≥ 5)	2.41 [1.16‐5.03]	.01	6.48 [1.65‐25.5]	.007	3.60 [1.69‐7.70]	.0001	3.95 [1.14‐13.7]	.02
Interaction terms								
Lung metastases × PROM2 IHC score ≥5			0.12 [0.02‐0.64]	.01				
Brain metastases × PROM2 IHC score ≥5							0.21 [0.04‐1.10]	.06

*Note*. Univariate and multivariate Cox proportional hazard regression models were run with the sample of deceased patients. The assumptions of the model were verified. Hazard ratios (HRs) for continuous variables were expressed per 1 SD or 1 interquartile range (IQR) as appropriate. Variables yielding *P*‐values under .2 in the univariate analysis were considered for inclusion in the multivariate analysis. A stepwise selection process of the lowest *P*‐values was used for the multivariate analysis, also using interaction terms. aHR: adjusted hazard ratio.

In this study, among patients with resectable regional lymph node metastases from cutaneous melanoma, we identified PROM2 as a biomarker significantly associated with the risk of distant metastases, particularly brain metastases, and a decreased survival. This association is strength of our study, with potential translational applications among patients with stage III melanoma. Indeed, adjuvant treatment using immunotherapy or targeted anti‐BRAF and anti‐MEK (anti‐Mitogen‐activated protein kinase) therapies is recommended for patients with stage III disease. No other marker than regional lymph node involvement is currently included in the therapeutic decision. After validation in a larger cohort, the “PROM2 IHC score” could be used to identify high‐ and low‐risk patients with stage III melanoma more efficiently, and could thus be included in adjuvant clinical trials.

In the management of metastatic melanomas, 20% to 40% of patients remain insensitive to targeted treatments.^9^, ^10^ Our study opens new perspectives for the use of PROM2 as a potential therapeutic target for the treatment of metastatic melanoma.

The role of PROM2 in the metastatic process has not been investigated, and further studies are required to elucidate this role and to see whether PROM2, like PROM1, provides stemness properties.

Our findings open new perspectives for further studies to validate PROM2 as a useful biomarker in adjuvant clinical trials, and as a potential biotarget for the treatment of metastatic melanoma in resort situations.

## ETHICS APPROVAL AND CONSENT TO PARTICIPATE

All patients had been informed that a part of their remaining samples could be used for research after diagnosis had been established, and none opposed it. Written informed consent was obtained from each patient. The Clinical Research Board Ethics Committee approved this study (CPP‐Ile‐de‐France#13218).

## CONFLICT OF INTEREST

The authors declare no conflict of interest.

## AUTHOR CONTRIBUTIONS

GB, AJ, and MB conceived and designed the study. PT, BB, JD, CL, and SM provided patient clinical data. MB provided patient tumor samples. TTN and MEB carried out the laser microdissections. GG and JPF analyzed the transcriptomic data. TTN and ChL performed the immunostaining. FP did statistical analyses. AJ, GB, and MB provided financial support. AJ and GB provided administrative support. TTN, GB, and MB drafted the manuscript. All authors read and approved the final version of the manuscript.

## Supporting information

Supporting information

Supporting information

Supporting information

Supporting information

Supporting information

Supporting information

Supporting information

Supporting information

Supporting information

Supporting information

Supporting information

## Data Availability

All data generated or analyzed during this study are included in this published article and its Supporting Information.
