# Supplementary material for: Increased risk of brain metastases among patients with melanoma and PROM2 expression in metastatic lymph nodes
Source: Clin Transl Med. 2020 Dec 2;10(8):e198. doi: 10.1002/ctm2.198 (PMC7711084; doi:10.1002/ctm2.198)
Supplement: Supplementary file 1 — Supporting information [file CTM2-10-e198-s001.docx]

**Supplementary Materials and Methods**

We have followed reporting recommendations for tumour marker prognostic studies (REMARK recommendations) ^1^.

**Patient data**

One hundred and one patients from Saint-Louis Hospital with available tumour samples and follow-up data were included in this study, for the development and the validation cohorts. Informed consent was obtained from each patient. The Clinical-Research-Board-Ethics-Committee approved this study (CPP-Ile-de-France#13218).

All patients were diagnosed between 2009 and 2014, and had a regional macroscopic lymph node metastatic melanoma without distant metastases at the time of inclusion in this study. None of them received systemic adjuvant therapy. Fifty-one patients were included in the development cohort, with samples collected between 2009 and 2013. Fifty additional patients were included in the validation cohort, with samples collected between 2013 and 2014.

On the basis of clinical and imaging data, and a median follow-up of 48 months from the time of the regional lymph node disease, patients with and without distant metastases were separated, including those with brain metastases. As recommended by the National Comprehensive Cancer Network, magnetic resonance imaging (MRI) of the brain was systematically performed at the time of the metastatic disease, and then at least once a year, or in case of neurological symptoms.

**Laser-microdissection of tumour cells from metastatic lymph nodes and transcriptomic data processing**For the development cohort, after consultation of the pathology department register (Saint-Louis Hospital), we identified 336 patients with melanoma and regional lymph node metastatic samples collected between 2009 and 2013. Among them, 100 patients had at least one frozen metastatic sample available and 58 patients had a follow-up of at least 36 months from diagnosis of metastatic disease (Supplementary Fig. 1). Cryo-cut sections of each sample were laser-micro-dissected to select tumor cells. Using a PALMMicrobeam/Zeiss-system, a minimum of 1500 tumor cells were laser-micro-dissected on 7 μm-thick tissue sections for a minimum surface area of 471 000 µm². Total RNA was extracted from the laser-micro-dissected tumor cells using RNeasy-Mini-Kit (Qiagen, France), it was quantified on NanoDrop and qualified on Bio-RadExperionTM Automated- Electrophoresis-Station (BioRad, France). Seven out of the 58 cases had RNA of insufficient quality. The remaining 51 cases had a mean RNA integrity number of 8.7 (range 7-10) (Supplementary Fig. 1).

Transcriptomic analyses were performed using MiltenyiBiotec-Microarray. A linear T7-based amplification step was performed on 0.5 μg of all RNA samples. To produce Cy3-labeled cRNA, the RNA samples were amplified and labelled using the Agilent-Quick-labelling kit. Yields of cRNA and dye-incorporation rates were measured with a ND-1000-Spectrophotometer

(NanoDrop, LabTech, France). Hybridization was performed according to the Agilent 60-mer-oligomicroarray protocol: 1.65 μg of Cy3-labeled cRNA were hybridized (overnight/65°C) on
Agilent-Whole-Human-Genome-Oligo-Microarrays 8 × 60K V2, and ﬂuorescence signals were detected using an AgilentMicroarray-Scanner. Agilent-FE-Software determined feature intensities. Quantile normalization was performed using the limma package on R-software version 3.2.1(Foundation for Statistical Computing, Vienna, Austria), based on log2 single-intensity expression data.

**RT-qPCR and validation of *PROM2* mRNA expression in metastatic lymph-nodes**

For the development cohort, on following sections of the same laser-micro-dissected metastatic lymph nodes, RT-qPCR was performed to validate the transcriptomic results for *PROM2* expression [Hs00376331_m1].

For the validation cohort, melanoma cancer cells from 50 metastatic lymph nodes were laser-micro-dissected and processed for RT-qPCR.

Total RNA was reverse-transcribed (cDNA) before qPCR amplification using random primers with SuperScriptTM-II-Reverse Transcriptase (Invitrogen, France). The qPCR reactions were performed using ﬂuorescent probes on a CFX96 Real-Time-System (Bio-Rad) according to the MIQE guidelines ^2^. A blank sample with no cDNA was included, and the experiments were performed in triplicate for each gene, each sample being duplicated on the PCR
plate. The reference gene TBP [Hs99999910_m1] was used to normalize gene expression results. The results were expressed as 2-ΔΔCq (relative quantification).

***In situ* PROM2 expression in metastatic lymph nodes**

Using immunochemistry, PROM2 expression was assessed in the metastatic lymph nodes of the 51 samples already processed for transcriptomic analyses, and of the 50 samples from the validation cohort. An indirect immuno-peroxidase method (Discovery/RocheDiagnostics) on 5µm-thick frozen tissue sections was used using anti-PROM2 (ab74997, rabbit polyclonal, Abcam, 1/100) as primary antibody, and anti-rabbit OmniMap detection kit (Roche-Diagnostics). Systematic controls were the absence of a primary antibody and the use of an irrelevant primary antibody of the same isotype. Normal skin and normal liver were used as positive and negative controls respectively.

For each tissue section, cells expressing PROM2 were counted by two different pathologists (MB, AJ) on five different fields at ×400 magnification, using a ProvisAX70 microscope (Olympus, Tokyo) with wide-field eyepiece number 26.5, providing a field size of 0.344mm2 at this magnification. The pathologists were blinded to clinical data.

A membranous and cytoplasmic distribution of PROM2 was considered positive. For each field, a minimum of 100 tumor cells were analysed. The percentage of PROM2-expressing cells was the number of positive cells among these 100 tumor cells.

Each sample was given a score by multiplying the stain intensity grade (0 = no staining, 1 = low intensity, 2 = medium intensity, 3 = strong intensity) by the numerical code for the percentage of positive cells (0 = 0%, 1 = under 10%, 2 = 10–50%, 3 = 51–80%, 4 = over 81%). The maximum score was 12 when more than 81% of the cells expressed PROM2 with a strong intensity signal. Results were expressed as mean ± standard error of the mean (SEM).

**Statistical analyses**

The data were analyzed using R statistical software (version 3.4.3, R Foundation for Statistical Computing, Vienna, Austria; <http://www.r-project.org>).

For descriptive analysis, categorical variables were summarized as the number (percentage), and continuous variables were summarized as the mean ± standard deviation (SD) or the median [interquartile range 25^th^-75^th^] as appropriate. “PROM2 IHC score” was categorized by the median of 5 as low (< 5) or high (≥ 5). Multivariate imputation by chained equations was used to handle missing data for *PROM2* expression (n=2), and “PROM2 IHC score” (n=10), via the MICE package in R.

*Transcriptomic data:* The Bioconductor Limma package was used to read Gene-expression-assay data files, to apply a background correction, to filter probes and to normalize gene expression across samples. The SAMR package was used to identify differentially expressed genes in samples with the two-class unpaired method, based on the ExpressionSet generated by the Limma package. An estimated *P*-value was reported for each gene, from a set of permutations in SAM analysis (n=1500 permutations). *P*-values were corrected for multiple comparisons using the false discovery rates (FDR) method. FDR analysis enables multiple comparisons of the *P*-value distribution, and calculates a threshold corresponding to a defined *Q*-value (0.001), where the parameter q represents the upper limit of the proportion of differentially expressed genes that are false positives. A minimum *Q*-value <0.001 was fixed, meaning that the differentially expressed gene list contained only 0.1% false positives. The score(d) or d-score represents the T-statistic value that measures the size of the difference relative to the variation in our sample data.

We performed the same analyses on transcriptomic data downloaded from public databanks. Transcriptomic data of metastatic melanomas were imported from TCGA and Gene Expression Omnibus (GEO) databases. "HT-seq counts" files of the TCGA SKCM cohort ^3^ were downloaded with the TCGAbiolinks R package and normalization was performed with the DESqe2 package, applying a variance stabilizing transformation to the count data. Processed data of the following GEO series were downloaded and analyzed: GSE22155 ^4^, GSE65904 ^5^, GSE60464 ^6^.

*Factors associated with brain metastases:* Patients with and without brain metastases at baseline were compared regarding their clinical characteristics (age, sex, primary site of melanoma, metastatic site, and the presence of ulceration), and *BRAF* status, *PROM2* expression and the intensity of “PROM2 IHC score” using Chi square test or Fisher’s exact test as appropriate for categorical variables and Student’s t-test or Wilcoxon’s test for quantitative variables as appropriate. Univariate and multivariate logistic regression were performed with the sample of brain metastatic patients. Odd ratios (ORs) for continuous variables were expressed per SD or interquartile range (IQR) as appropriate. Variables yielding *P*-values under 0.2 in the univariate analysis were considered for inclusion in the multivariate analysis. A backward elimination process of the highest *P*-values was used for the multivariate analysis. Variables associated with brain metastatic patients in multivariate analysis were expressed as an adjusted OR (aOR) [95% confidence interval (CI)].

*Factors associated with mortality:* Univariate survival curves were plotted according to the Kaplan-Meier method for brain metastatic status, and “PROM2 IHC score”. Univariate and multivariate Cox proportional hazard regression were performed with the sample of patients deceased. The assumptions of the model were verified. Hazard ratios (HRs) for continuous variables were expressed per SD or interquartile range (IQR) as appropriate. Variables yielding *P*-values under 0.2 in the univariate analysis were considered for inclusion in the multivariate analysis. A backwards elimination process of the highest *P*-values was used for the multivariate analysis. Variables associated with mortality in multivariate analysis were expressed as an adjusted HR (aHR) [95%CI]. We checked potential interaction terms between all predictors of survival to adjust the final multivariate model.

**References**

**1.** McShane LM, Altman DG, Sauerbrei W, et al. REporting recommendations for tumour MARKer prognostic studies (REMARK). *British journal of cancer.* 2005; 93(4):387-391.

**2.** Bustin SA, Benes V, Garson JA, et al. The MIQE guidelines: minimum information for publication of quantitative real-time PCR experiments. *Clin Chem.* 2009; 55(4):611-622.

**3.** Guan J, Gupta R, Filipp FV. Cancer systems biology of TCGA SKCM: efficient detection of genomic drivers in melanoma. *Sci Rep.* 2015; 5:7857.

**4.** Jonsson G, Busch C, Knappskog S, et al. Gene expression profiling-based identification of molecular subtypes in stage IV melanomas with different clinical outcome. *Clin Cancer Res.* 2010; 16(13):3356-3367.

**5.** Cirenajwis H, Ekedahl H, Lauss M, et al. Molecular stratification of metastatic melanoma using gene expression profiling: Prediction of survival outcome and benefit from molecular targeted therapy. *Oncotarget.* 2015; 6(14):12297-12309.

**6.** Jilaveanu LB, Parisi F, Barr ML, et al. PLEKHA5 as a Biomarker and Potential Mediator of Melanoma Brain Metastasis. *Clin Cancer Res.* 2015; 21(9):2138-2147.
