# Supplementary material for: Increased risk of brain metastases among patients with melanoma and PROM2 expression in metastatic lymph nodes
Source: Clin Transl Med. 2020 Dec 2;10(8):e198. doi: 10.1002/ctm2.198 (PMC7711084; doi:10.1002/ctm2.198)
Supplement: Supplementary file 2 — Supporting information [file CTM2-10-e198-s002.docx]

**Supplementary Figure 1**

Diagram for selection criteria of the 51 patients of the development cohort.

**Supplementary Figure 2**

Survival curves in two cohorts of melanoma lymph node metastases from public databanks, according to *PROM2* mRNA expression level (high or low).

1. TCGA SKCM cohort (reference 13 in the manuscript)
2. GSE22155 and GSE65904 cohorts (references 14 and 15 in the manuscript)

**Supplementary Figure 3**

Survival curves in the validation cohort, according to the presence or not of brain metastases (upper panel), or to the “PROM2 IHC score” (lower panel). The presence of brain metastases and/or a “PROM2 IHC score” ≥ 5 are significantly associated with a shorter survival.
