# Supplementary figures and images for: Increased risk of brain metastases among patients with melanoma and PROM2 expression in metastatic lymph nodes
Source: Clin Transl Med. 2020 Dec 2;10(8):e198. doi: 10.1002/ctm2.198 (PMC7711084; doi:10.1002/ctm2.198)

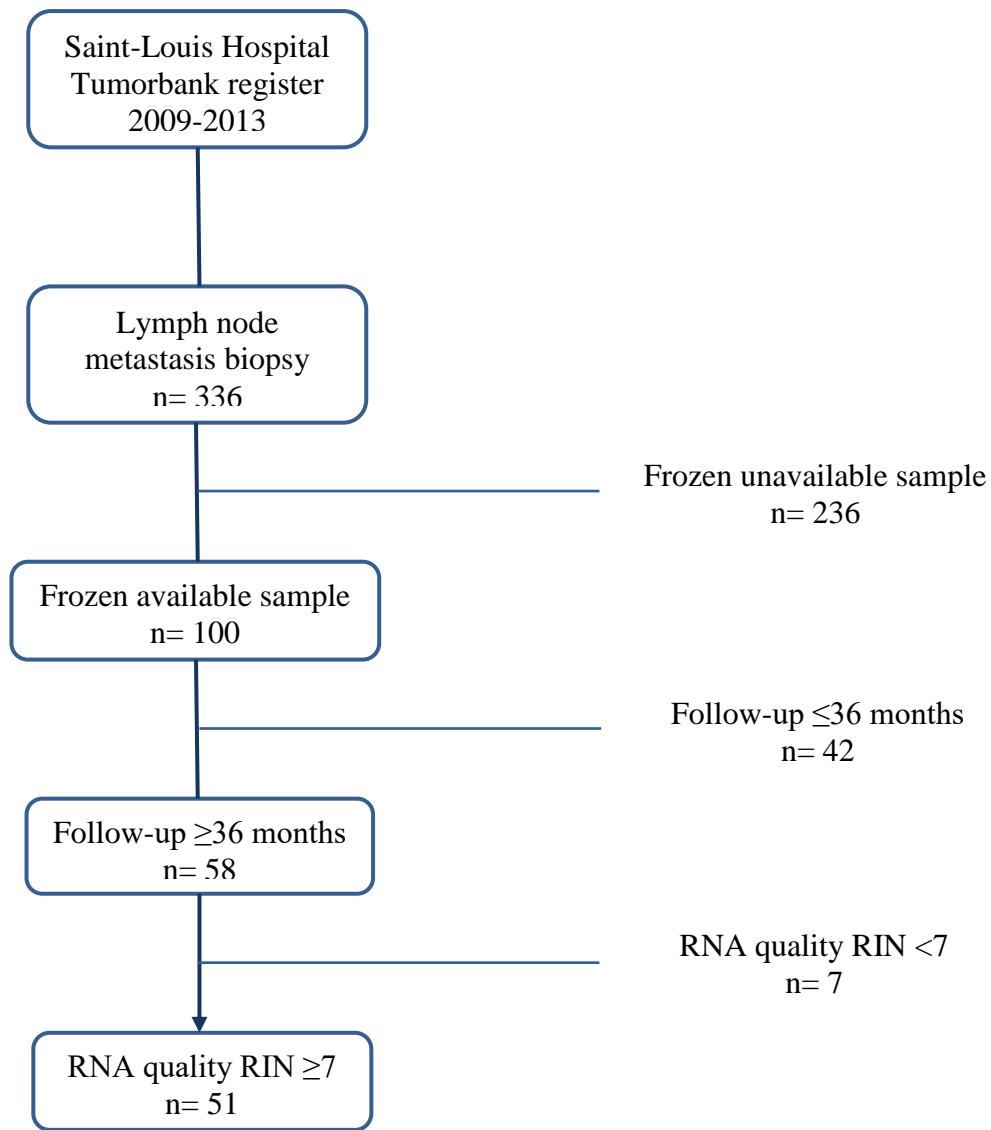

**Supplementary Figure 1.**

Supplement: Supplementary file 3 — Supporting information [file CTM2-10-e198-s003.pdf]

**A**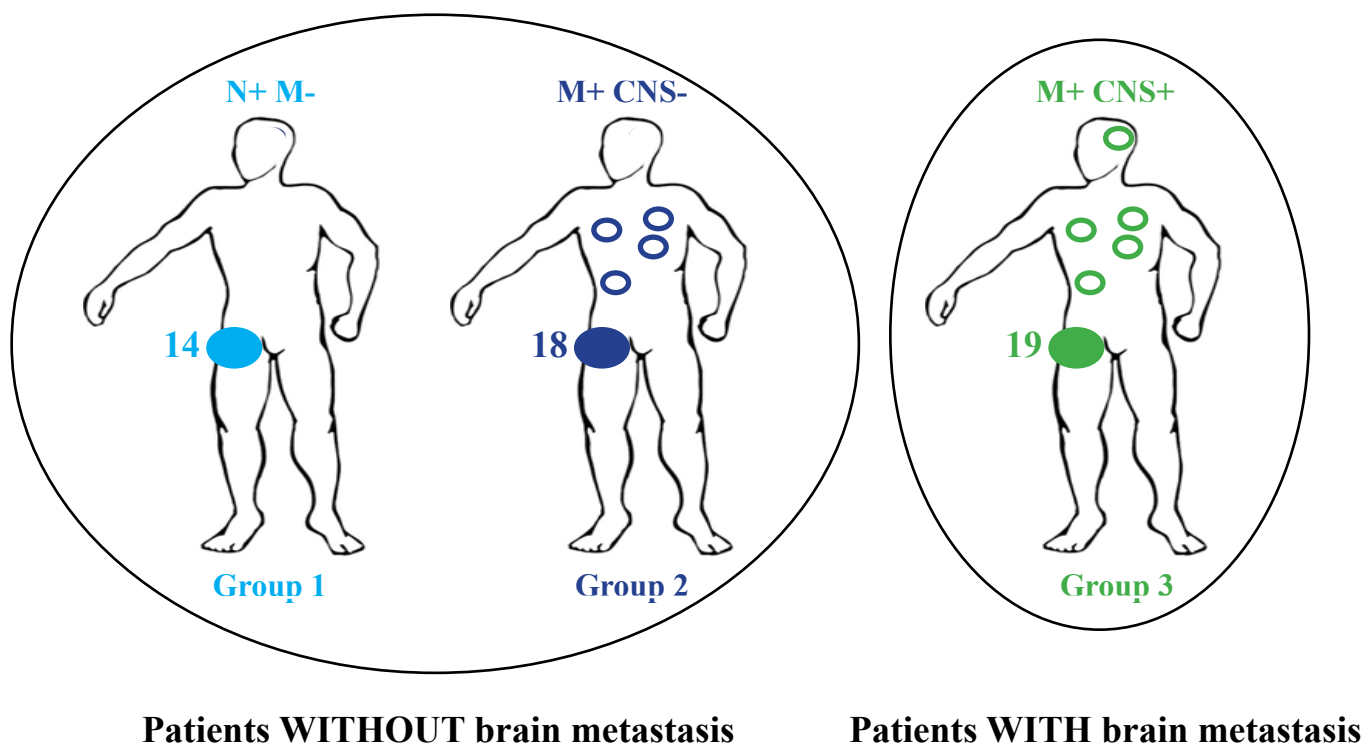**B**

Series of 51 patients with transcriptomic analyses

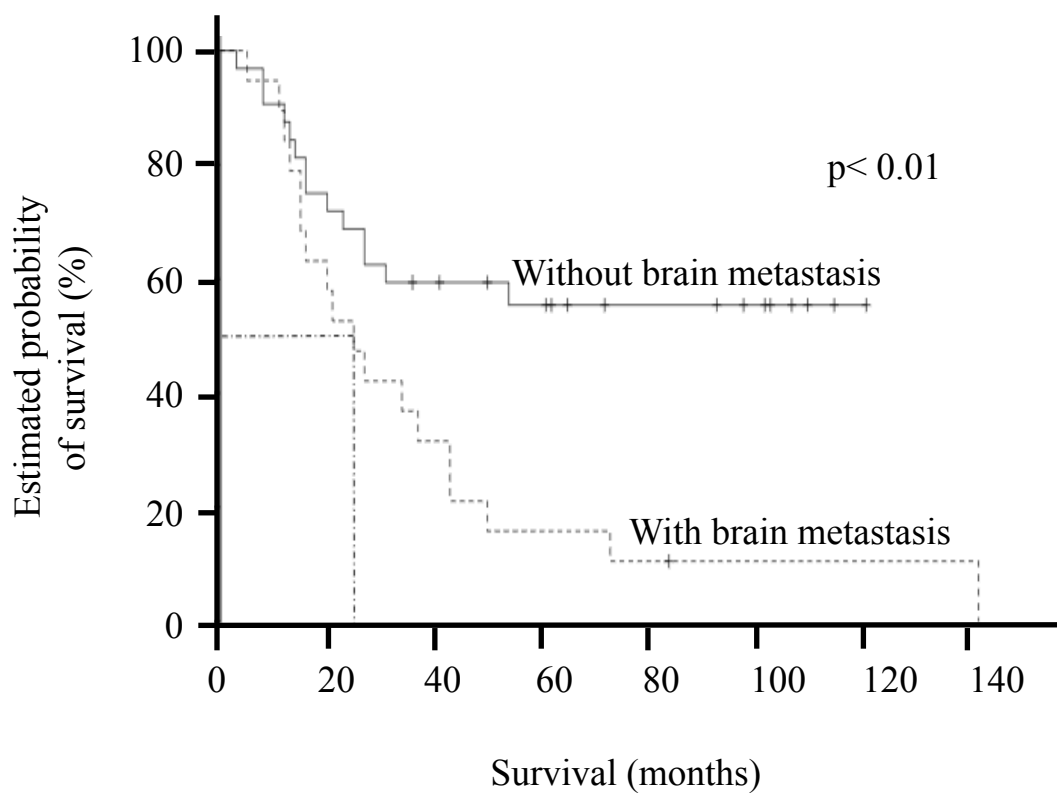

Supplement: Supplementary file 4 — Supporting information [file CTM2-10-e198-s004.pdf]

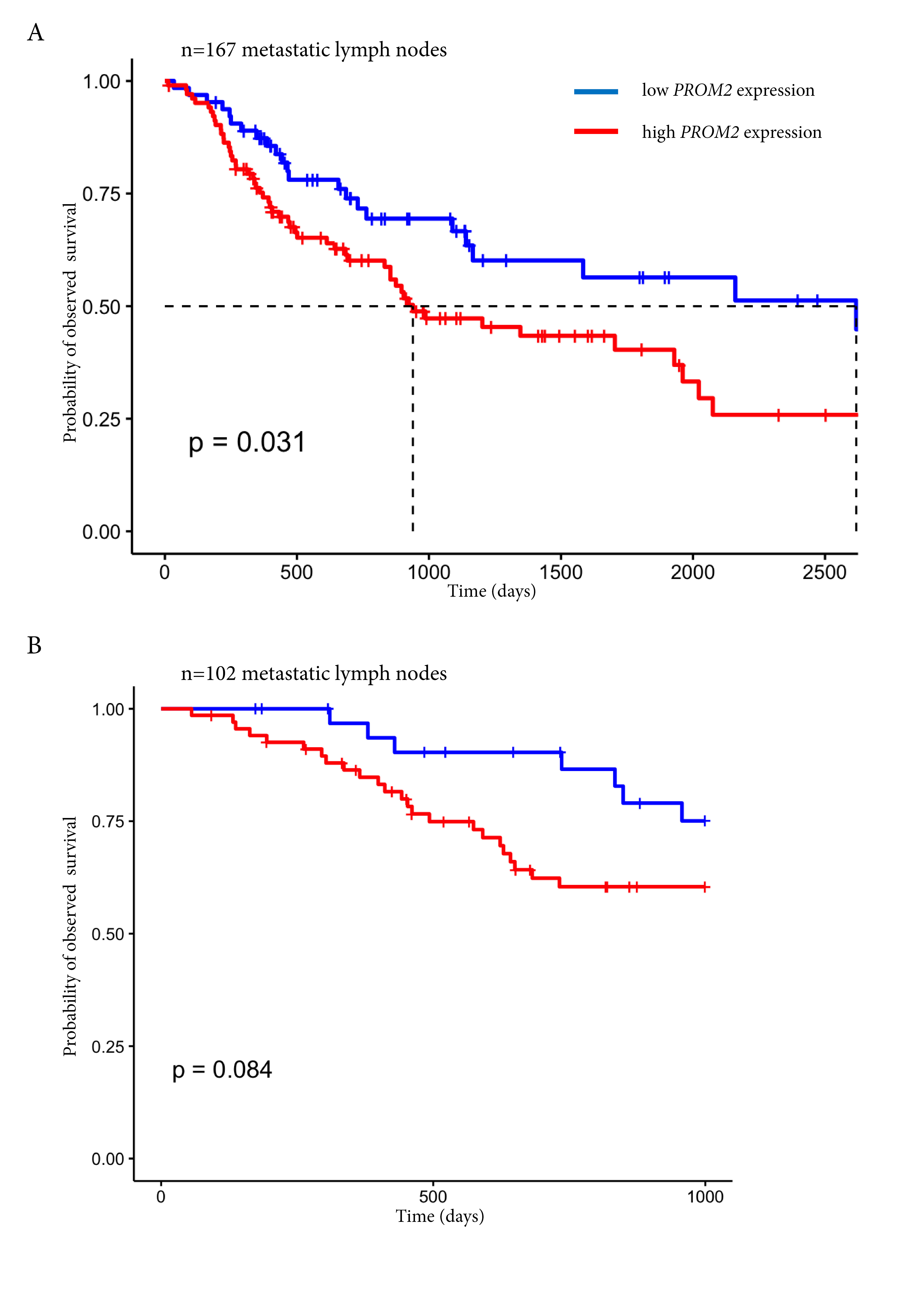

Supplement: Supplementary file 5 — Supporting information [file CTM2-10-e198-s005.jpg]

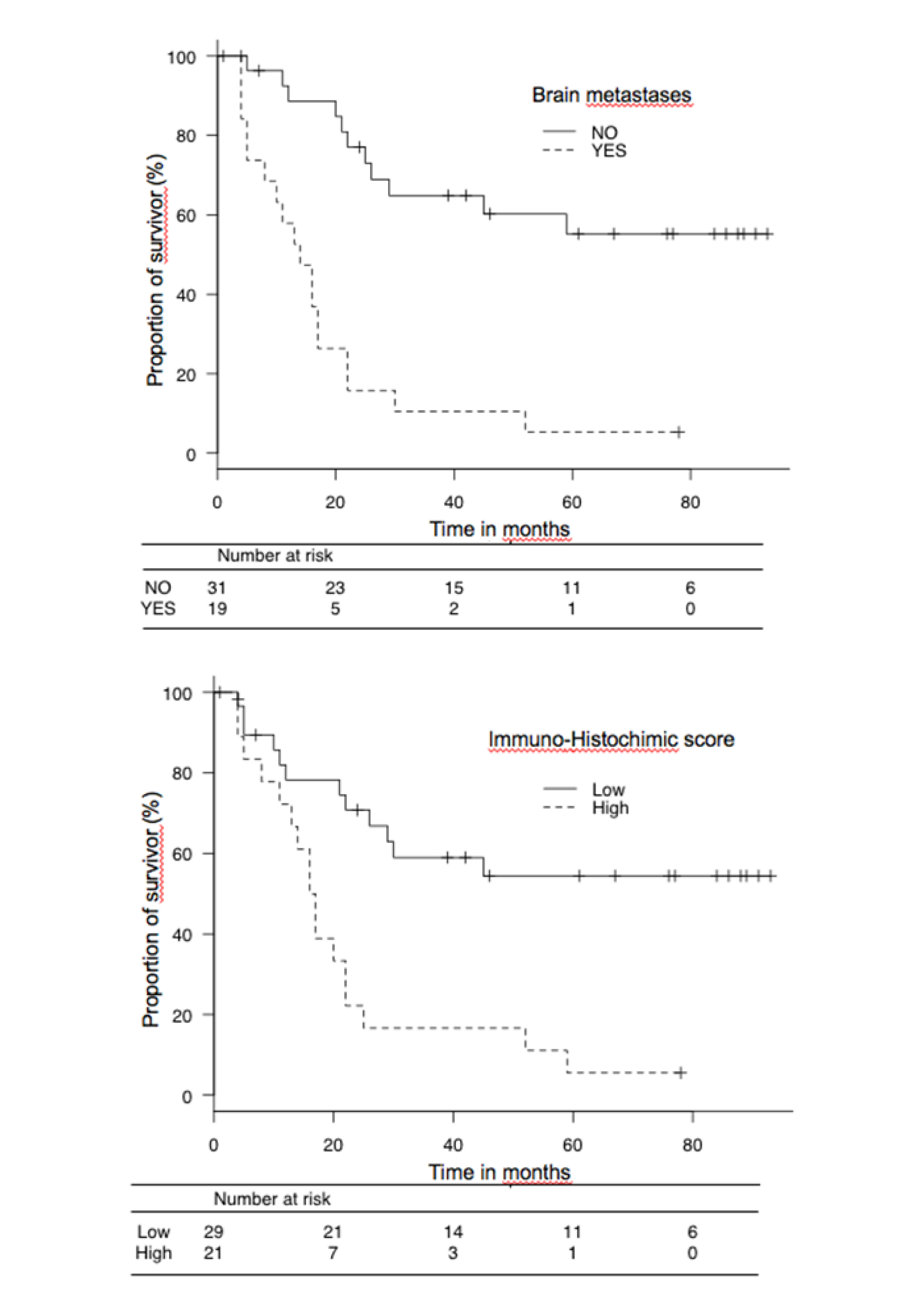

Supplement: Supplementary file 6 — Supporting information [file CTM2-10-e198-s006.jpg]
