# Supplementary material for: Increased risk of brain metastases among patients with melanoma and PROM2 expression in metastatic lymph nodes
Source: Clin Transl Med. 2020 Dec 2;10(8):e198. doi: 10.1002/ctm2.198 (PMC7711084; doi:10.1002/ctm2.198)
Supplement: Supplementary file 7 — Supporting information [file CTM2-10-e198-s007.doc]

Supplementary Table 1. Characteristics of the 51 patients in the development cohort with transcriptomic analyses of metastatic lymph nodes

| **Variables** | **N (%)** | **Brain metastases (Group 3)**  **N=19 (37%)** | **No brain metastasis**  **(Group 1 and 2)**  **N=32 (63%)** | ***P**** |
| --- | --- | --- | --- | --- |
| Age (y), mean ± SD | 57.8 ± 15.8 | 55.0 ± 15.6 | 59.5 ± 15.9 | 0.3 |
| Gender (women) | 28 (55) | 11 (58) | 17 (53) | 0.7 |
| Initial TNM classification^#^:  IIIB  IIIC  IIID | 20 (39)  28 (55)  3 (6) | 7 (37)  10 (53)  2 (10) | 13 (41)  18 (56)  1 (3) | 0.5 |
| Primary site of melanoma:  Head and neck  Trunk  Upper limb  Lower limb  Non-available | 5 (10)  10 (20)  11 (21)  23 (45)  2 (4) | 3 (16)  3 (16)  5 (26)  8 (42)  0 | 2 (6)  7 (22)  6 (19)  15 (47)  2 (6) | 0.6 |
| Metastatic site:  Lung  **Bone**  Liver | 24 (47)  **11 (21)**  16 (31) | 12 (63)  **8 (42)**  8 (42) | 12 (37.5)  **3 (9)**  8 (25) | 0.07  **0.01**  0.2 |
| Breslow index (mm), median (25^th^-75^th^) | 3.5 (2.0-5.0) | 3.25 (2.01-7.0) | 3.58 (1.42-4.87) | 0.4 |
| Ulceration (yes) | 18 (41) | 8 (42) | 10 (31) | 0.7 |
| *BRAF* status:  *BRAF V600E*  Wild type | 26 (51)  25 (49) | 9 (47)  10 (53) | 17 (53)  15 (47) | 0.7 |

^#^ TNM classification according to 2017 AJCC 8^th^ edition

* Chi square test or Fisher’s exact test as appropriate for categorical variables and Student’s t-test or Wilcoxon’s test for quantitative variables as appropriate
