# Supplementary material for: Increased risk of brain metastases among patients with melanoma and PROM2 expression in metastatic lymph nodes
Source: Clin Transl Med. 2020 Dec 2;10(8):e198. doi: 10.1002/ctm2.198 (PMC7711084; doi:10.1002/ctm2.198)
Supplement: Supplementary file 8 — Supporting information [file CTM2-10-e198-s008.doc]

Table 2: Genes differentially expressed in metastatic lymph nodes of patients with *versus* without brain metastasis

| **Gene** | **Without brain metastasis (mean)** | **With brain metastasis (mean)** | **Fold  Change** | **d-score** | ***P*-value** |
| --- | --- | --- | --- | --- | --- |
| **PROM2** | **5,7** | **7,5** | **3,3** | **4.6** | **2.1e-05** |
| LRRC2 | 7,4 | 9,3 | 3,6 | 4.2 | 2.2e-05 |
| CASC15 | 9,8 | 11,7 | 3,6 | 4.2 | 2.2e-05 |
| HMBOX1 | 7,2 | 9,0 | 3,5 | 4.1 | 2.2e-05 |
| GPR179 | 5,9 | 7,7 | 3,4 | 4.3 | 2.2e-05 |
| CD86 | 9,6 | 11,4 | 3,4 | 4.0 | 2.3e-05 |
| RPA4 | 7,1 | 8,8 | 3,2 | 4.3 | 2.1e-05 |
| GPR182 | 5,8 | 7,5 | 3,1 | 4.4 | 2.1e-05 |
| KRT79 | 5,1 | 6,7 | 2,9 | 3.9 | 2.3e-05 |
| PPP6R1 | 8,0 | 9,4 | 2,7 | 3.7 | 2.5e-05 |
| BICC1 | 5,1 | 6,5 | 2,6 | 4.1 | 2.2e-05 |
