# Supplementary material for: Increased risk of brain metastases among patients with melanoma and PROM2 expression in metastatic lymph nodes
Source: Clin Transl Med. 2020 Dec 2;10(8):e198. doi: 10.1002/ctm2.198 (PMC7711084; doi:10.1002/ctm2.198)
Supplement: Supplementary file 9 — Supporting information [file CTM2-10-e198-s009.doc]

**Table 3. Univariate and multivariate analyses of factors associated with brain metastasis**

| **Variables** | **Univariate analysis**  **OR [95%CI]** | ***P*** | **Multivariate analysis**  **Adjusted OR [95%CI]** | | | ***P*** |
| --- | --- | --- | --- | --- | --- | --- |
| Metastatic site:  Lung  **Bone** | 2.86 [0.88-9.25]  7.03 [1.57-31.4] | 0.08  **0.01** | | 1.41 [0.27-7.27]  16.2 [1.83-395.9] | 0.66  **0.02** | |
| PROM2 expression, per 1 IQR | 1.21 [1.03-1.48] | **0.03** | | 1.05 [0.91-1.36] | 0.62 | |
| **PROM2 IHC score:**  Low (< 5)  High (≥ 5) | 1 (reference)  16.0 [4.10-83.1] | **0.0002** | | 1 (reference)  28.2 [4.33-570.7] | **0.003** | |
|  | | | | | | |
