# Supplementary material for: Increased risk of brain metastases among patients with melanoma and PROM2 expression in metastatic lymph nodes
Source: Clin Transl Med. 2020 Dec 2;10(8):e198. doi: 10.1002/ctm2.198 (PMC7711084; doi:10.1002/ctm2.198)
Supplement: Supplementary file 10 — Supporting information [file CTM2-10-e198-s010.doc]

**Supplementary Table 1. Baseline characteristics and comparison between the development and the validation cohort**

| **Variables** | **Development cohort**  **n = 51** | **Validation cohort**  **n = 50** | ***P**** |
| --- | --- | --- | --- |
| Age (y), mean ± SD | 58 ± 16 | 63 ± 16 | 0.11 |
| Gender (women) | 28 (55) | 20 (40) | 0.14 |
| Initial TNM classification:  IIIB  IIIC  IIID | 20 (39)  28 (55)  3 (6) | 23 (46)  25 (50)  2 (4) | 0.73 |
| Primary site of melanoma:  Head and neck  Trunc  Upper extremity  Lower extremity  Non-available | 5 (10)  10 (20)  11 (21)  23 (45)  2 (4) | 4 (8)  17 (34)  9 (18)  19 (38)  1 (2) | 0.59 |
| Metastatic site at relapse:  Brain  Lung  Bone  Liver | 19 (37)  24 (47)  11 (21)  16 (31) | 19 (38)  19 (38)  12 (24)  17 (34) | 0.94  0.36  0.77  0.78 |
| Breslow index (mm), median (IQR) | 3.5 (3.0) | 2.5 (2.7) | 0.27 |
| Ulceration (yes) | 18 (41) | 21 (42) | 0.34 |
| BRAF status:  BRAF V600E | 26 (51) | 27 (54) | 0.25 |
| * Chi square test or Fisher’s exact test as appropriate for categorical variables and Student’s t-test or Wilcoxon’s test for quantitative variables as appropriate.  IQR: interquartile range. | | | |
