# Supplementary material for: Increased risk of brain metastases among patients with melanoma and PROM2 expression in metastatic lymph nodes
Source: Clin Transl Med. 2020 Dec 2;10(8):e198. doi: 10.1002/ctm2.198 (PMC7711084; doi:10.1002/ctm2.198)
Supplement: Supplementary file 11 — Supporting information [file CTM2-10-e198-s011.doc]

**Supplementary Table 2. Univariate analyses of an association between a high “PROM2 IHC score” ≥5 and other metastatic sites than brain metastases**

| **Types of metastases** | **Development cohort**  **OR [95%CI]** | ***P*** | **Validation cohort**  **Adjusted**  **OR [95%CI]** | | | ***P*** |
| --- | --- | --- | --- | --- | --- | --- |
| Lung  Bone  Liver | 2.38 [0.77-7.34]  1.47 [0.38-5.60]  1.71 [0.52-5.65] | 0.13  0.57  0.37 | | 4.19 [1.25-14.1]  0.61 [0.16-2.41]  0.65 [0.19-2.19] | **0.02**  0.48  0.49 | |
|  | | | | | | |
